# Aortic arch branch-prioritized reconstruction for type A aortic dissection surgery

**DOI:** 10.3389/fcvm.2023.1321700

**Published:** 2024-01-23

**Authors:** Jianfeng Gao, Jie Yan, Yanyu Duan, Junjian Yu, Wentong Li, Zhifang Luo, Wenbo Yu, Dilin Xie, Ziyou Liu, Jianxian Xiong

**Affiliations:** ^1^ The First Clinical Medical College, Gannan Medical University, Ganzhou, China; ^2^ Department of Thoracic Surgery, The First People’s Hospital of Nankang District, Ganzhou, China; ^3^ Heart Medical Centre, First Affiliated Hospital of Gannan Medical University, Ganzhou, China; ^4^ Engineering Research Center of Intelligent Acoustic Signals of Jiangxi Province, Key Laboratory of Prevention and Treatment of Cardiovascular and Cerebrovascular Diseases, Ministry of Education, Gannan Medical University, Ganzhou, China; ^5^ Ganzhou Cardiovascular Rare Disease Diagnosis and Treatment Technology Innovation Center, Gannan Medical University, Ganzhou, China; ^6^ Department of Cardiovascular Surgery, First Affiliated Hospital of Gannan Medical University, Ganzhou, China

**Keywords:** Stanford type A aortic dissection, branch priority, total arch replacement, frozen elephant trunk, surgery

## Abstract

**Background:**

Acute Stanford type A aortic dissection (STAAD) is a fatal condition requiring urgent surgical intervention. Owing to the complexity of the surgical process, various complications, such as neurological disorders, are common. In this study, we prioritized the reconstruction of aortic arch branches during surgery and investigated the association between prioritizing the branches and the postoperative outcomes of patients with STAAD.

**Methods:**

Ninety-seven patients were included in the observational study and underwent total arch replacement and frozen elephant trunk technique between January 2018 and June 2021. Of these, 35 patients underwent the branch-priority technique, and 62 patients underwent the classic technique. By analyzing the perioperative outcomes, we compared the differences between the two techniques.

**Results:**

The branch priority group had significantly shorter cardiopulmonary bypass and ventilator times and earlier postoperative wake-up times than the classic group. Additionally, the ICU stay time was shorter, with a significant decrease in neurological complications and 24 h drainage in the branch priority group compared to the classic group.

**Conclusion:**

The branch priority technique can effectively provide better brain protection, resulting in earlier awakening of patients after surgery, reduced neurological complications, shorter ventilation time and decreased ICU hospitalization time. Therefore, it is recommended for use in aortic dissection surgeries.

## 1 Introduction

Acute Stanford type A aortic dissection (STAAD) is a catastrophic cardiovascular disease with an extremely high mortality rate during the acute phase. Surgery is the preferred treatment for patients with STAAD (1). Total arch replacement and frozen elephant trunk (FET) technique are standard surgical procedures for STAAD. Complex surgery requires a skilled cardiac surgical team to maintain stable blood pressure throughout the perioperative period, particularly, to prevent sudden changes in blood pressure that could result in dissection rupture before the surgery. Close cooperation among surgeons, anesthesiologists, and cardiopulmonary bypass (CPB) specialists during surgery is equally important.

However, owing to the inherent characteristics of the STAAD surgery, it requires longer CPB and cardiac arrest times than other cardiac surgeries. As well as selective cerebral perfusion during the surgery and the effects of deep hypothermia and circulatory arrest, postoperative complications, such as bleeding, hypoxemia, and acute renal insufficiency, are more likely to occur. Neurological complications are relatively common, and the surgical mortality rate is still high (2, 3). Aortic surgeons are dedicated to enhancing surgical techniques and brain protection methods to minimize neurological complications. These include improvements in stitching techniques, sequence of vascular reconstruction, cerebral perfusion strategies, and intraoperative temperature management (4–6).

In this study, we adjusted the order of vascular reconstruction and prioritized aortic arch branch reconstruction technique for STAAD surgery. This technique requires establishing a bypass from the right femoral artery to the right axillary artery at the start of the surgery. Reconstruction of the arch branch, including the innominate artery (IA), the left common carotid artery (LCCA), and the left subclavian artery (LSCA), was first performed without CPB. This study aimed to observe the application of branch priority technology in STAAD surgery to determine whether it has certain advantages and whether it can effectively prevent postoperative neurological complications in patients.

## 2 Methods

This retrospective study was approved by the Ethics Committee of the First Affiliated Hospital of Gannan Medical University (Number LLSC2023,163) and was exempt from obtaining the patients’ personal consent.

### 2.1 Patients

This retrospective study included patients with STAAD who underwent total arch replacement and FET technique at our hospital between January 2018 and June 2021. The exclusion criteria were as follows: (1) Patients with hemodynamic instability requiring urgent establishment of CPB; (2) Patients requiring simultaneous aortic valve replacement or mitral valve surgery, or requiring root reconstruction (Bentall procedure), or coronary artery bypass grafting (CABG), as these procedures significantly increased CPB time and cardiac blockade times, resulting in a higher mortality rates; (3) Patients receiving anticoagulant drugs or platelet inhibitors before surgery, which caused coagulation dysfunction and difficulty in hemostasis, leading to increased intraoperative blood loss and postoperative drainage. Ultimately, 97 patients were included in this study. Among them, 35 underwent the branch-priority technique, whereas 62 underwent the classic technique. The data collected included general information such as age, sex, body mass index (BMI), left ventricular ejection fraction (LVEF), aortic regurgitation, mitral regurgitation, ascending aortic diameter, pericardial effusion, creatinine (Cr), and troponin I (cTnI), as well as intraoperative and postoperative data such as CPB time, aorta clamp time, circulation arrest time, and nasopharyngeal temperature. ICU stay time, ventilator time, postoperative dialysis, transfusion of red blood cells, plasma, and platelets, and wake-up time. Neurological complications were defined as the occurrence of any of the following conditions: (I) postoperative delirium confirmed through CAM-ICU method evaluation (7); (II) transient nerve injury (focal neurological deficits diagnosed through transient CT or MRI); and (III) permanent neurological dysfunction (the occurrence of new focal injuries, systemic dysfunction, or multiple brain injuries after surgery).

### 2.2 Surgical procedure

#### 2.2.1 Branch priority group

**Figure 1:**
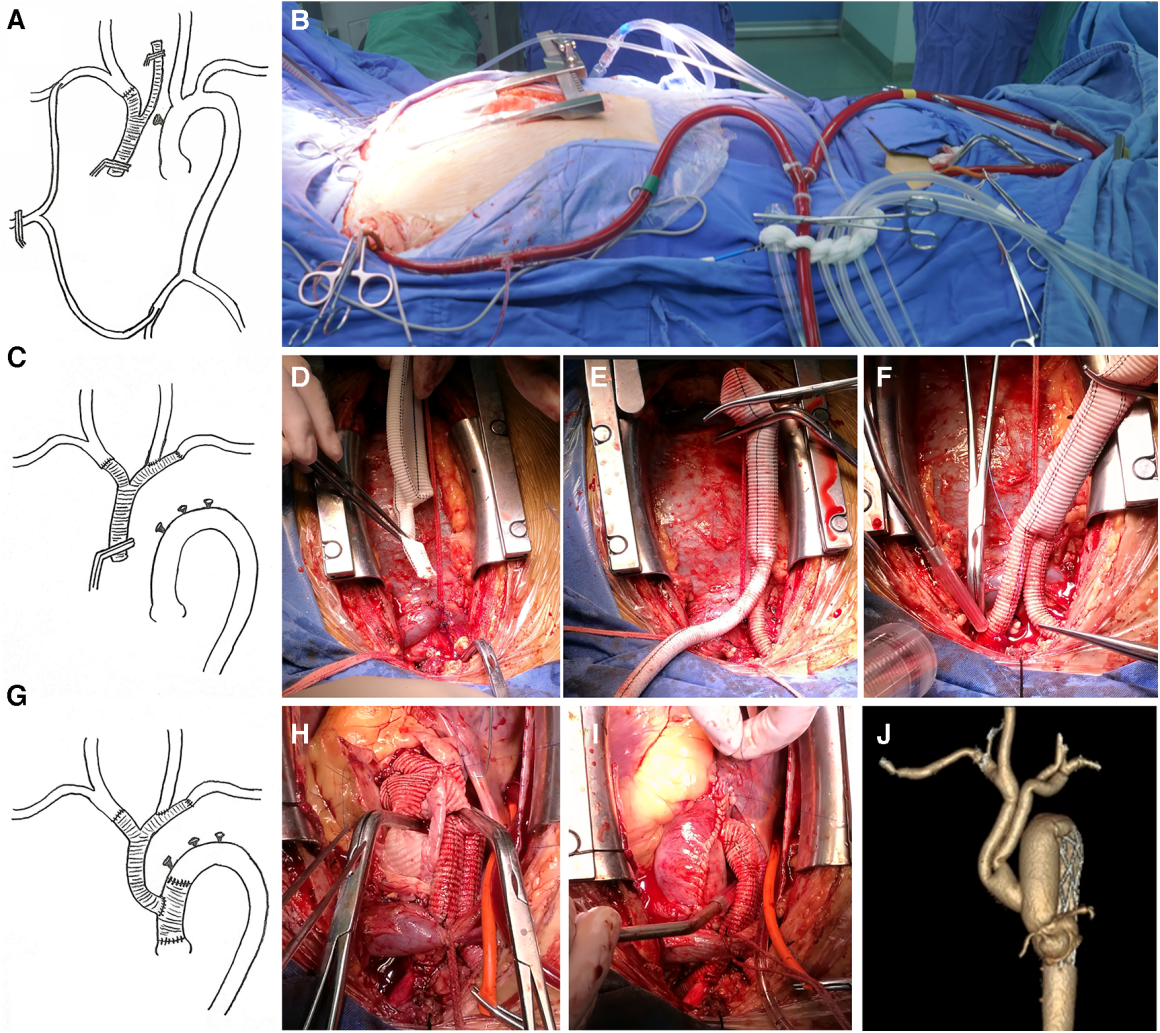
(**A,B**) The right axillary artery and the right common femoral artery are cannulated separately, and the two cannulas are connected through an arterial perfusion catheter of the CPB. The bypass from the right femoral artery to the axillary artery is thus established, (**D–F**). The UA, LSCA, and LCCA are sequentially reconstructed by supplying blood to the brain through the bypass, (**G–I**). After completing the replacement surgery of the ascending aorta, the Y-shaped graft is anastomosed end-to-side with the ascending aortic graft, (**J**). Postoperative CTA imaging.

The branch priority group used a Y-shaped graft (MAQUET double velvet artificial blood vessel 16 × 9 × 9) to reconstruct the arch branches. A sternal median incision was made to expose the innominate artery (IA), left common carotid artery (LCCA), and left subclavian artery (LSCA). The right axillary artery and the right common femoral artery were cannulated separately, and the two cannulas were connected using an arterial perfusion catheter for CPB. This established a bypass from the right femoral artery to the axillary artery (Figures 1A,B). When the IA is blocked, blood flow to the right carotid artery can be restored using the right femoral artery as a bypass. The IA was cut off, and the proximal end was sutured, and anastomosed with a 9 mm branch of the Y-shaped graft (Figures 1C,D). Another 9 mm branch was used to anastomose with the LSCA, Then, a hole was drilled on the side wall of the graft branch and anastomosed with the LCCA (Figures 1E,F). After completing the arch branch reconstruction, CPB was established by cannulating the right atrium. Antegrade infusion of histidine-tryptophan-ketoglutarate was used for myocardial protection. The aortic root was reconstructed using an appropriately size graft. When the patient's temperature drops to about 28 °C, the perfusion flow of CPB is adjusted to approximately 10 ml/kg/min and the femoral artery cannula is clamped. During circulatory arrest, global brain perfusion is maintained by the right axillary artery, while maintaining a pressure of 40–60 mmHg in the left radial artery. Total arch replacement and FET technique are performed on the aortic arch. The ascending aorta was clamped, and systemic perfusion was restored through femoral artery perfusion. The 16 mm main trunk of the Y-shaped graft is trimmed to an appropriate angle and length, and it is anastomosed end-to-side with the ascending aorta graft (Figures 1G–J).

#### 2.2.2 Classic group

CPB was established through the right axillary artery and right atrial cannulation, and HTK was applied for myocardial protection. A tetrafurcate graft was used for root reconstruction at the proximal end, and FET was performer at the distal aortic arch. The selective cerebral perfusion method was used during circulatory arrest. Systemic perfusion was restored once the FET procedure was completed. Then, the arch branches are reconstructed during CPB, which requires a lower nasopharyngeal temperature (25°C) to ensure brain protection.

### 2.3 Statistical analysis

Continuous variables were expressed as mean and standard deviation or median and quartiles, as appropriate, and further analyzed using either the Student's *t*-test or the Mann–Whitney *U*-test. Categorical variables were analyzed using the chi-squared test or Fisher's exact test. Statistical significance was set at *p* < 0.05 significant.

## 3 Results

### 3.1 Preoperative characteristics

Preoperative characteristics are listed in Table 1. No statistically significant differences were observer in sex, age, BMI, cardiac function (LVEF), ascending aortic diameter, renal function (Cr), cTnI (ug/l), valve function (AR and MR), or hemopericardium between the two groups.

**Table 1:** Preoperative characteristics.

	Classic group (*n* = 62)	Branch prioritized group (*n* = 35)	*t*	*P*
Male, *n* (%)	43 (69.4)	23 (65.7)	–	0.712
Age, year, M ± SD	66.4 ± 7.6	67.9 ± 8.9	0.804	0.424
BMI*, Kg/m2, M ± SD	27.8 ± 7.5	26.4 ± 7.2	0.820	0.415
LVEF*, %, M ± SD	60.4 ± 4.7	59.4 ± 4.9	0.908	0.767
AD*, cm, M ± SD	43.4 ± 12.9	46.4 ± 8.3	1.129	0.262
Cr*, μmol/l, M ± SD	69.5 ± 5.9	71.3 ± 6.1	1.305	0.196
cTnI*, ug/l, M ± SD	0.2 ± 1.0	0.1 ± 0.2	0.531	0.597
AR*, *n* (%)	35 (56.5)	17 (48.6)	–	0.456
MR*, *n* (%)	19 (30.6)	10 (28.6)	–	0.830
Hemopericardium, *n* (%)	8 (12.9)	5 (14.3)	–	1.000

### 3.2 Operative data

The operative data are presented in Table 2. No statistically significant differences (*P* > 0.05) were observer in aortic clamp time, circulation arrest time, or intraoperative blood transfusion. Patients in the branch priority group had significantly shorter CPB time (187.4 ± 38.3 vs. 208.5 ± 61.3 min; *P* < 0.05) and higher nasopharyngeal temperature (28.1 ± 10.1 vs. 23.9 ± 1.2°C; *P* < 0.05).

**Table 2:** Operative data and postoperative outcomes.

	Classic group (*n* = 62)	Branch priority group (*n* = 35)	*t*	*P*
CPB* time, min, M ± SD	208.5 ± 61.3	187.4 ± 38.3	3.274	0.002
Aorta clamp time, min, M ± SD	115.3 ± 10.3	112.3 ± 12.8	1.228	0.222
Circulation arrest time, min, M ± SD	25.6 ± 4.6	26.4 ± 7.2	0.613	0.542
Nasopharyngeal temperature, °C, M ± SD	23.9 ± 1.2	28.1 ± 10.1	16.035	0.000
Intraoperative RBC transfusion, U, M ± SD	5.0 ± 1.2	4.8 ± 1.3	1.174	0.605
Intraoperative plasma transfusion, ml, M ± SD	476.3 ± 121.1	523.5 ± 136.6	1.162	0.111
Intraoperative platelet transfusion, U, M ± SD	1.2 ± 0.8	1.3 ± 0.7	0.565	0.574

### 3.3 Postoperative outcomes

The postoperative outcomes are shown in Table 3. There was no statistically significant difference (*P* > 0.05) in renal failure requiring dialysis, secondary thoracotomy for hemostasis, tracheotomy, low cardiac output, and death during hospitalization. Patients in the branch priority group had significantly shorter earlier wake-up time (4.1 ± 1.3 vs. 6.3 ± 5.6 h; *P* < 0.05) and ventilator time (17.5 ± 8.1 vs. 35.8 ± 13.6 h; *P* < 0.05) compared to the classic group. The ICU stay time was also shorter (24.3 ± 1.2 vs. 51.1 ± 15.6 h; *P* < 0.05), and a significant reduction in neurological complications [2 (5.7%) vs. 13 (21.0%); *P* < 0.05] and 24 h drainage (491.5 ± 87.4 vs. 731.5 ± 137.5 ml; *P* < 0.05) was observed.

**Table 3:** Postoperative outcomes.

	Classic group (*n* = 62)	Branch priority group (*n* = 35)	*t*	*P*
ICU stay time (h)	51.1 ± 15.6	24.3 ± 1.2	8.333	0.000
Ventilator time/h	35.8 ± 13.6	17.5 ± 8.1	6.621	0.000
Wake-up time/h	6.3 ± 5.6	4.1 ± 1.3	2.080	0.041
Secondary thoracotomy	3 (4.8)	2 (5.7)	–	1.000
24 h drainage/ml	731.5 ± 137.5	491.5 ± 87.4	8.496	0.000
Dialysis	6 (9.7)	3 (8.6)	–	1.000
Tracheotomy	2 (3.2)	1 (2.9)	–	1.000
Low cardiac output	3 (4.8)	2 (5.7)	–	1.000
Neurological complications	13 (21.0)	2 (5.7)	–	0.046
Hospitalization death	2 (3.2)	1 (2.9)	–	1.000

### 3.4 One-year follow-up outcomes

The 1-year follow-up outcomes are presented in Table 4. No statistically significant differences were observed in one-year mortality rates. Stenosis at the brachiocephalic vascular anastomosis was defined as a stenosis rate > 50%. The classic group includes one patient with stenosis at the LCCA anastomosis and one at the LSCA anastomosis. The two patients experienced dizziness caused by cerebral hypoperfusion, which was resolved by interventional stent implantation, leading to improved symptoms.

**Table 4:** One-year follow-up outcomes.

	Classic group (*n* = 62)	Branch priority group (*n* = 35)	*P*-value
Mortality at follow-up, *n* (%)			
30-day	2 (3.2)	1 (2.9)	1.00
3 months	3 (4.8)	1 (2.9)	1.00
6 months	3 (4.8)	2 (5.7)	1.00
12 months	4 (6.5)	2 (5.7)	1.00
Emerging neurological disorders	2 (3.2)	0 (0)	0.538
Anastomotic stenosis			
IA	0 (0)	0 (0)	1.00
LCC	1 (1.6)	0 (0)	1.00
LSCA	1 (1.6)	0(0)	1.00

## 4 Discussion

STAAD is one of the most fatal diseases in the cardiovascular system. Conservative treatment is associated with a significant mortality risk. Patients often succumb to organ dysfunction resulting from aortic dissection rupture, pericardial tamponade, inadequate blood supply to the aortic branches (such as acute myocardial infarction and intestinal ischemic necrosis), acute renal insufficiency, and other complications (8, 9). The mortality rate is approximately 50% within 48 h of onset and 70% within one month. Surgery is the preferred treatment method, and classic total arch replacement and FET techniques are currently the standard surgical methods (10, 11). However, owing to the complexity of the procedure, longer surgical and CPB times are required because of numerous suture operations. Additionally, the significant impact of deep hypothermia on coagulation function increases intraoperative blood loss and necessitates more red blood cell and platelet transfusions (12, 13). Furthermore, implementing effective brain protection measures during surgery is challenging for the surgical procedure and postoperative recovery. Currently, there is no clear definition in the academic community regarding whether intraoperative brain protection involves selective or bilateral cerebral perfusion (14). Neurological complications are a common phenomenon after surgery (15). Postoperatively, delayed wakefulness, vision problems, and strokes often occur. This poses a huge challenge for the cardiac surgery team that had just performer STAAD surgery.

Since 2018, our hospital has performed surgeries on patients with STAAD. In the initial stages, we found that a high occurrence of postoperative neurological dysfunction and hospital mortality led to a low success rate in treating patients with STAAD. At the same time, bleeding is one of the unavoidable challenges in aortic dissection surgery (16). These include surgical bleeding caused by inexperienced suture techniques and coagulation dysfunction resulting from factors such as low temperature or improper use of anticoagulants before surgery. Therefore, we are committed to improving surgical procedures and suture techniques, providing enhanced methods for brain protection, and reducing the risk of intraoperative bleeding. We aimed to raise the intraoperative temperature to minimize coagulation function damage and shorten CPB times. These factors are of great significance in ensuring the success of surgery. Therefore, while gaining surgical experience, we implemented branch-priority technology for eligible patients.

Dr. Straueh and Dr. Spielvogel are pioneers in the branch priority technique. They were the first to adopt a bilateral cerebral perfusion strategy for better brain protection (17, 18). However, the intraoperative core body temperature was still maintained at deep hypothermia (15°C), which significantly impaired coagulation function.

We gradually developed and refined branch-prioritized reconstruction technique for aortic arch surgery in patients with STAAD. First, a bypass was established between the right femoral artery and the axillary artery. This was performed during the reconstruction of the brachiocephalic artery, and it allowed for the brain's blood supply to be provided by bypass from the autologous circulation. A technique that does not require CPB during brachiocephalic artery reconstruction can reduce CPB time. In this study, patients in the branch priority group had a significantly shorter CPB time than those in the classic group (187.4 ± 38.3 vs. 208.5 ± 61.3 min; *P* < 0.05). Subsequent CPB involves the simultaneous perfusion of the femoral and axillary arteries. The combination of cannulation perfusion in the right axillary and femoral arteries ensures control of blood flow during CPB. This approach benefits patients with high body weight or a smaller right axillary artery, as it decreases complications resulting from inadequate organ perfusion. Global cerebral perfusion can be ensured when brachiocephalic artery reconstruction is completed. This ensures clear brain protection during subsequent hypothermic circulatory arrest, and the temperature can be increased to 28°C during circulatory arrest. Our study showed that the branch priority group had an earlier wake-up time (4.1 ± 1.3 vs. 6.3 ± 5.6 h; *P* < 0.05) and shorter ventilator time (17.5 ± 8.1 vs. 35.8 ± 13.6 h; *P* < 0.05) compared to the classic group. The ICU stay time was also shorter in the branch priority group than in the classic group (24.3 ± 1.2 vs. 51.1 ± 15.6 h; *P* < 0.05).

After he brachiocephalic artery reconstruction, the operating space of the aortic arch increased significantly. This increase is beneficial for anastomosis of the arch and reduces the time pressure on surgeons during subsequent operations. Consequently, this helps ensure the quality of the anastomosis and reduces the risk of anastomotic bleeding.

Y-shaped grafts (MAQUET) have readily available products that are easy to obtain. A 16 mm main stem and 9 mm branches, can provide ample blood supply to the brachiocephalic artery. Arch branch prioritized reconstruction can provide bilateral cerebral perfusion during the subsequent hypothermic arrest, significantly reducing nervous system damage. This study showed that patients in the branch-priority group had better postoperative outcomes, including reduced neurological complications and earlier wake-up time than those in the classic group.

However, not all STAAD surgeries can adopt this branch-priority technique. Therefore, it is necessary to evaluate whether the perfusion of the femoral artery is affected by aortic dissection before surgery. If this is the case, effective blood perfusion from the femoral artery to the axillary artery cannot be achieved, even with the establishment of a temporary bypass. If the blood flow in the femoral artery is not affected by dissection and the implementation of branch-priority technology is successful, it is necessary to evaluate the safety of cerebral perfusion. This can be achieved by monitoring the blood flow of the bypass and using near-infrared spectroscopy to observe cerebral oxygen saturation (19). It is worth noting that when using branch-priority technology, the perfusion flow during circulatory arrest may be greater than that of traditional selective cerebral or bilateral cerebral perfusion flow because the LSCA receives perfusion simultaneously.

Aortic dissection may cause the intercostal artery to be supplied by the false lumen, leading to severe spinal cord ischemia and paraplegia (20–22). In Sun's procedure, circulatory arrest can lead to temporary spinal cord ischemia, which should also be taken seriously. Lowering core body temperature can improve the spinal cord's tolerance to ischemia. The branch-priority technique can provide bilateral cerebral and bilateral subclavian artery perfusion, effectively supplying partial blood to the spinal cord during circulatory arrest. It has been reported that there is an increased risk of spinal cord ischemia if temperatures exceed 28°C during circulatory arrest. Additionally, excessive circulatory arrest time (>40 min) prolongs the duration of spinal cord ischemia (23). Our branch prioritizes technology that sets a nasopharyngeal temperature of 28°C for patients during circulatory arrest. In this study, the average nasopharyngeal temperature in the priority-group was 27.0 ± 1.0°C, which was significantly higher than that in the classic group (23.8 ± 1.1°C, *P* < 0.001). Regarding the current cases and their subsequent outcomes, no complications related to paraplegia in the branch priority group were observed.

## 5 Conclusions

In summary, compared with the classic Sun's procedure, the branch priority technique can shorten CPB time, ensure bilateral cerebral perfusion throughout the surgery, reduce the occurrence of neurological complications, enable patients to wake up earlier after surgery, and promote better patient recovery. The length of ICU stay was also reduced.

## Data Availability

The raw data supporting the conclusions of this article will be made available by the authors, without undue reservation.

## References

[1] Rylski B Schilling O Czerny M. Acute aortic dissection: evidence, uncertainties, and future therapies. Eur Heart J. (2023) 44(10):813–21. 10.1093/eurheartj/ehac757 36540036

[2] Carrel T Sundt TM 3rd von Kodolitsch Y Czerny M. Acute aortic dissection. Lancet. (2023) 401(10378):773–88. 10.1016/S0140-6736(22)01970-5 36640801

[3] Ferreira A Oliveira-Pinto J Mansilha A. Mortality and neurologic complications after repair of aortic arch pathology with elephant trunk procedures: a systematic review with meta-analysis. Int Angiol. (2022) 41(5):372–81. 10.23736/S0392-9590.22.04924-0 36053160

[4] Jabagi H Juanda N Nantsios A Boodhwani M. Aortic arch surgery at 32°C: mild hypothermia and unilateral antegrade cerebral perfusion. Interact Cardiovasc Thorac Surg. (2021) 32(5):773–80. 10.1093/icvts/ivaa321 33432355 PMC8691572

[5] Benedetto U Dimagli A Cooper G Uppal R Mariscalco G Krasopoulos G Neuroprotective strategies in acute aortic dissection: an analysis of the UK national adult cardiac surgical audit. Eur J Cardiothorac Surg. (2021) 60(6):1437–44. 10.1093/ejcts/ezab192 33963362 PMC8643475

[6] Cao L Guo X Jia Y Yang L Wang H Yuan S. Effect of deep hypothermic circulatory arrest versus moderate hypothermic circulatory arrest in aortic arch surgery on postoperative renal function: a systematic review and meta-analysis. J Am Heart Assoc. (2020) 9(19):e017939. 10.1161/JAHA.120.017939 32990132 PMC7792363

[7] Keenan CR Jain S. Delirium. Med Clin North Am. (2022) 106(3):459–69. 10.1016/j.mcna.2021.12.003 35491066

[8] Harris KM Nienaber CA Peterson MD Woznicki EM Braverman AC Trimarchi S Early mortality in type A acute aortic dissection: insights from the international registry of acute aortic dissection. JAMA Cardiol. (2022) 7(10):1009–15. 10.1001/jamacardio.2022.2718 36001309 PMC9403853

[9] Ma L Chai T Yang X Zhuang X Wu Q Chen L Outcomes of hemi- vs. Total arch replacement in acute type A aortic dissection: a systematic review and meta-analysis. Front Cardiovasc Med. (2022) 9:988619. 10.3389/fcvm.2022.988619 36237909 PMC9552831

[10] Bashir M Mohammed I Al-Tawil M Jubouri M Agbobu T Chen EP. Frozen elephant trunk: the gold standard. Cardiovasc Diagn Ther. (2023) 13(3):623–7. 10.21037/cdt-23-144 37405021 PMC10315428

[11] Iino K Takago S Saito N Ueda H Yamamoto Y Kato H Total arch replacement and frozen elephant trunk for acute type A aortic dissection. J Thorac Cardiovasc Surg. (2022) 164(5):1400–9.e3. 10.1016/j.jtcvs.2020.10.135 33341270

[12] Jeppesen AN Duez C Kirkegaard H Grejs AM Hvas AM. Fibrinolysis in cardiac arrest patients treated with hypothermia. Ther Hypothermia Temp Manag. (2023) 13(3):112–9. 10.1089/ther.2022.0037 36473198

[13] Wu J Qiu J Fang Z Luo Q Huang Y Yu C Optimal degree of hypothermia in total arch replacement for type A aortic dissection. Front Cardiovasc Med. (2021) 8:668333. 10.3389/fcvm.2021.668333 33996953 PMC8115724

[14] Tasoudis PT Varvoglis DN Vitkos E Ikonomidis JS Athanasiou T. Unilateral versus bilateral anterograde cerebral perfusion in acute type A aortic dissection repair: a systematic review and meta-analysis. Perfusion. (2023) 38(5):931–8. 10.1177/02676591221095468 35575301

[15] Pitts L Kofler M Montagner M Heck R Iske J Buz S Cerebral protection strategies and stroke in surgery for acute type A aortic dissection. J Clin Med. (2023) 12(6):2271. 10.3390/jcm12062271 36983272 PMC10056182

[16] Lee JH. Prevention and management of difficult hemostasis in acute type A aortic dissection repair. Asian Cardiovasc Thorac Ann. (2023) 31(1):15–9. 10.1177/02184923221074409 35040355

[17] Strauch JT Spielvogel D Lauten A Galla JD Lansman SL McMurtry K Technical advances in total aortic arch replacement. Ann Thorac Surg. (2004) 77(2):581–89; discussion 9–90. 10.1016/S0003-4975(03)01342-0 14759442

[18] Spielvogel D Strauch JT Minanov OP Lansman SL Griepp RB. Aortic arch replacement using a trifurcated graft and selective cerebral antegrade perfusion. Ann Thorac Surg. (2002) 74(5):S1810–4; discussion S25–32. 10.1016/S0003-4975(02)04156-5 12440671

[19] Zhou X Xia Y Uchitel J Collins-Jones L Yang S Loureiro R Review of recent advances in frequency-domain near-infrared spectroscopy technologies [invited]. Biomed Opt Express. (2023) 14(7):3234–58. 10.1364/BOE.484044 37497520 PMC10368025

[20] Wang L Zhong G Lv X Dong Y Hou Y Chen L. Clinical outcomes of mild versus moderate hypothermic circulatory arrest with antegrade cerebral perfusion in adult aortic arch surgery: a systematic review and meta-analysis. Perfusion. (2022):2676591221144169. 10.1177/02676591221144169. [Epub ahead of print] 36476142

[21] Tsai YD Hsu CW Hsu CC Liao WI Chen SJ. Paraplegia caused by aortic coarctation complicated with spinal epidural hemorrhage. Am J Emerg Med. (2016) 34(3):680.e1–2. 10.1016/j.ajem.2015.06.057 26275629

[22] Tan L Xiao J Zhou X Shen K Li F Luo J Untreated distal intimal tears may be associated with paraplegia after total arch replacement and frozen elephant trunk treatment of acute Stanford type A aortic dissection. J Thorac Cardiovasc Surg. (2019) 158(2):343–50.e1. 10.1016/j.jtcvs.2018.08.111 30396731

[23] Leontyev S Borger MA Etz CD Moz M Seeburger J Bakhtiary F Experience with the conventional and frozen elephant trunk techniques: a single-centre study. Eur J Cardiothorac Surg. (2013) 44(6):1076–82; discussion 83. 10.1093/ejcts/ezt252 23677901

